# In Memoriam—I. T. Talbot, M. D.

**Published:** 1899-09

**Authors:** 


### IN MEMORIAM—I. T. TALBOT, M. D.

At a meeting of the Committee appointed by the President of the American Institute of Homœopathy to draft resolutions on the death of Dr. Israel Tisdale Talbot, the following were presented and adopted:

In accordance with the inexorable law which governs all created things, our colleague and ex-President of the American Institute of Homœopathy, Israel Tisdale Talbot, M. D., has been called to rest from his labors; therefore

*Resolved,* That we deplore the loss of one who, having the deepest interest in the cause of Homœopathy, had done more than any other member to insure the growth and success of this Institute. Possessing great executive ability, eminently gifted in the organization and government of large bodies, to him this Institute is indebted for its admirable constitution and code of by-laws.

We shall miss him at our gatherings as he was rarely absent from our meetings, miss his words of counsel, his matured judgment in all matters appertaining to the furtherance of this body, miss his cordial greeting and his interest in each individual.

He could truly say, "I have fought a good fight, I have finished my course. I have kept the faith." We are confident "that henceforth there is laid up for him a crown of righteousness."

*Resolved,* That the American Institute of Homœopathy extends to his widow and family the deepest sympathy in their great bereavement; that these resolutions be entered on our record, and a copy be transmitted to his family.

Henry E. Spalding, M. D.,
Hiram L. Chase, M. D.,
Conrad Wesselhœft, M. D.,
Adeline B. Church, M. D.,
Frank C. Richardson, M. D.,
*Committee.*

